# Neuropragmatics: from classical pragmatics to neurocognitive models of pragmatics in dialogue

**DOI:** 10.3389/fpsyg.2026.1745034

**Published:** 2026-03-17

**Authors:** Angélica Gutiérrez Cisneros, Alice Foucart, Angèle Brunellière

**Affiliations:** 1 Univ. Lille, CNRS, UMR 9193 - SCALab - Sciences Cognitives et Sciences Affectives, F-59000, Lille, France; 2 Centro de Investigación Nebrija en Cognición (CINC) & Facultad de Lenguas y Educación, Universidad Antonio de Nebrija, Madrid, Spain; 3 Univ. Lille, Inria, CNRS, Centrale Lille, UMR 9189 - CRIStAL, F-59000, Lille, France

**Keywords:** cognitive approach, neural substrates, neurocognitive models, neuropragmatics, speech acts, temporal dynamics

## Abstract

Over the past 30 years, there has been significant development in the understanding of the brain mechanisms underlying pragmatic processing. The primary purpose of the present review is to delve into the origins of neuropragmatics, defined as the study of the neural basis of pragmatic processing, tracing its development from the foundations of traditional pragmatics. Moreover, this review aims to deepen the understanding of the cognitive and neural mechanisms underlying pragmatic processing. Throughout this review, the topic of neuropragmatics is addressed through diverse theoretical frameworks modeling how the pragmatic information is treated. Then, the neural substrates and neurophysiological correlates of pragmatic processing are outlined, with particular interest in the study of speech acts which emerged more recently at the brain level. Lastly, we discuss promising directions to address the questions that remain unresolved in the field of neuropragmatics, which have huge impacts on methodological and societal aspects.

## 1 Introduction

A key element when studying language is analyzing the context in which it occurs, as context can completely change the meaning of the message. For example, if someone says: “What a lovely weather!” the sentence does not mean the same thing when uttered during a warm, sunny day as it does during a cold, rainy one. Likewise, a speaker’s intention when replying “Playing Vivaldi is complicated” is not the same when responding to “How hard is it to play Vivaldi?” as when responding to “Did you like what I played?” the latter implying that the piece was not well received. This is where *pragmatics* comes to be: the study of language in context, of the circumstances in which it is used, and of the relationships between the meaning of words and the intentions of the speaker. As demonstrated by Alduais et al.’s (2025) bibliometric and scientometric review, over the past 20 years, there has been substantial growth in the empirical data of pragmatic processing. A precise understanding of neuropragmatics therefore requires tracing its evolution from the foundations of traditional pragmatics to current neurocognitive research. The intersection of philosophical-linguistic and cognitive approaches is addressed in the first two sections of this review, to explain the emergence of neuropragmatics. To add up to past reviews in neuropragmatics (Bambini, 2010; Bambini and Bara, 2012; Bara and Tirassa, 2000; Bischetti et al., 2024; Domaneschi and Bambini, 2020; Hagoort and Levinson, 2014) that have effectively defined the scope of the field, linking it to clinical research, identifying its basic neural infrastructure, and addressing the temporal dynamics of pragmatic processing, the present review provides a thorough account of how the field developed from philosophical-linguistic approaches. Moreover, it compares current models of neuropragmatics by detailing their contributions and limitations, and it provides insight into how pragmatic processing unfolds spatio-temporally in the brain. Crucially, this review highlights the need for an integrative theoretical framework that incorporates the perspectives from current models of neuropragmatics and is capable encompassing the variability inherent to pragmatic processing. Finally, the review outlines emerging research directions within the field to address unresolved gaps and inform on the development of future models and frameworks.

## 2 From classical pragmatics to neuropragmatics

### 2.1 Defining classical pragmatics of philosophical-linguistic approaches

Classical “far-side” pragmatics is concerned with studying what is interpreted beyond what is “literally” said, to determine what information is conveyed or action performed in a given socio-linguistic context (Korta and Perry, 2024). Accordingly, while the literal meaning of a message is constructed using grammatical and lexical knowledge, pragmatic inferences allow the interpretation of the meaning conveyed by a speaker. Thus, far-side pragmatics clearly distinguishes between what is said and what the speaker implies, suggests, or means. Moreover, this distinction between what a person says and means intrinsically calls for a description of the circumstances in which the utterance is made, such as the speaker’s identity or the time of the utterance (Grice, 1968, 1975).

One of the most prominent language philosophers to pioneer far-side pragmatics was Grice (1968). He introduced the “Cooperative principle” (Grice, 1975), which postulates that when people communicate, they are “conversationally cooperative” denoting that they work together to achieve a shared communicative goal, namely a mutually accepted direction of their exchange in which they are engaged. This goal determines what conversational actions are suitable, guided by the four conversational maxims. These maxims are principles that speakers generally follow in communicative situations, including providing enough information (*quantity* maxim), ensuring it is true (*quality* maxim), pertinent (*relation* maxim), and clear (*manner* maxim). However, speakers often *flout* these maxims by saying one thing but intending another. Breaking a maxim is rarely accidental but rather deliberate, given that the speaker expects the listener to understand nonetheless, by inferring the intended meaning. This, in turn, gives rise to what Grice called a “conversational implicature,” in which a person says *p*, but implicates *q*. Thus, in order for a hearer to resolve a conversational implicature, they not only rely on the cooperative principle and the resulting maxims, but also on the background knowledge of the situation, the linguistic and extralinguistic context, and the conventional sense of the utterance. Hence, while semantics (i.e., meaning of words) deals exclusively with conventional meaning, pragmatics is concerned with the study of implicatures. Furthermore, Grice’s implicatures are inherently linked to the communicative intentions behind the utterance, that is the speaker meaning. These intentions are always oriented toward an addressee: the speaker expects, first, that the addressee will recognize and understand these intentions, and second, that they will respond accordingly. This expected response has been referred to as “Perlocutionary” act, as described in Austin’s (1962) work.

Parallel to Grice’s work another philosopher, Austin (1962), made foundational contributions to far-side pragmatics by developing the Speech act theory. This theory was finalized and published by his student Searle (1964), after Austin’s premature passing. Austin described Speech acts, also called “Locutionary acts,” as performing an act of saying something, which comprises three different types of acts: a phonetic act (concerning the noises uttered), a phatic act (pertaining to the grammatical structuring of words), and a rhetic act (regarding the meaning or sense of the utterance). Beyond Locutionary acts, Austin explained that in *saying* something, we parallelly *do* something else: an “Illocutionary” act, which conveys a specific force of Illocution, such as giving a warning, or making a statement. The resulting effect on the hearer (such as being persuaded, or misled) is in turn called Perlocutionary act, as described earlier.

Searle further described speech acts as being the “basic unit of linguistic communication” (Searle, 1964), governed by a set of *constitutive* rules established by a language’s structure. He stated that the Illocutionary force of an utterance is determined by “function indicating devices” such as, in English, the punctuation, stress, intonation, word order, and, most importantly, the context of the speech situation. Moreover, Searle reformulated Grice’s account of meaning (which states that the meaning of something said is more than just its literal sense), to comprise both an intentional and a conventional dimension of meaning, as well as the relationship between both (Searle, 1964, 1975).

### 2.2 Shifting towards cognitive approaches to pragmatics

From the philosophical-linguistic approaches of the 1960’s and 1970’s, pragmatics gradually evolved through models of communication that were increasingly psychologically plausible. Accordingly, in the mid-eighties, Sperber and Wilson’s Relevance theory adopted a cognitive approach to pragmatics, rather than a formal one, seen as it was concerned with both the results of the comprehension process, as well as the mental mechanisms that enable them (Wilson, 2019). The foundational work of the Relevance theory is rooted in a Gricean view of pragmatics, in which the basic feature of human communication is to express and recognize intentions, essentially, an inferential model of communication. However, Sperber and Wilson questioned Grice’s Cooperative principles and the use of maxims. They proposed the “Cognitive Principle of Relevance,” according to which relevance plays a crucial role not only in communication, but in human cognition overall (Wilson and Sperber, 2007). In any given utterance, the addressee has to assume that what people say is relevant. Utterances (incoming information) and internal mental representations (prior knowledge) serve as inputs to elaborate assumptions of relevance. The relevance of any input is then determined by how assumptions and the correct interpretation of the utterance interact to produce new “cognitive effects,” which correspond to the updates of the listener’s assumptions resulting from the integration of the inputs into their previous knowledge. In a communicative context, comprehension is guided by relevance: the listener seeks to construct the most informative interpretation of an utterance with the least cognitive effort (Noveck and Sperber, 2004; Wilson, 2019).

Sperber and Wilson’s cognitive turn in pragmatic theory helped pave the way for philosophers like Kasher to introduce the notion of “cognitive pragmatics” Kasher (1994). Contemporary research on discourse and dialogue comprehension (e.g., Graesser et al., 1997; Kintsch and van Dijk, 1978) provided cognitive approaches of inference generation and meaning construction that later informed cognitive-pragmatic reasoning. Nevertheless, it was not until 1999, with the publication of Bara’s (1999) monograph “Cognitive Pragmatics,” that the notion was formally established (Schmid, 2012). For Bara (2016), the “cognitive” component relies on the way that mental states ensure efficiently the comprehension of speakers’ intentions and the generation of communicative acts. Bara’s (2010) vision agreed with Grice on the idea that people cooperate when they communicate, given that they jointly construct the meaning of each exchange. However, it differed from Grice’s proposal by arguing that individuals can have different goals and mutual recognition of mental states between interlocutors is a prerequisite for successful communication. These mental states play an important role in producing and comprehending communication acts. Correspondingly, the elaboration of mental states is based upon three fundamental concepts of *cooperation*, *sharedness*, and *communicative intent*. The first element, *cooperation*, was introduced by Grice (1975). Then, based on the notion of “common ground,” described by Clark (1996) as a collection of beliefs, knowledge and suppositions that interlocutors share during a conversation, Bara (2016) stated sharedness is the second component for successful communication. However, in order to communicate efficiently, interlocutors are thought to require awareness of what they are sharing, called a meta-sharedness. Finally, the last concept for efficient communication is *communicative intent*. Bara (2016) resumed Grice’s proposal of first- and second-order intentions, namely the intent to achieve an effect on the interlocutor and said interlocutor recognizing this intent.

### 2.3 Bridging experimental and clinical methods: the birth of neuropragmatics

Pragmatics, being rooted in linguistics and philosophy of language, had mainly produced theories based on analyses of verbal examples coming from spoken exchanges or written texts and interpretation intuitions. Inspired by the methodologies from psycholinguistic and cognitive psychology research on verbal communication, such as reaction times and priming paradigms, the eighties and nineties witnessed an experimental shift in pragmatics marked by the pioneering research of Clark (1979), Clark and Lucy, 1975), Gibbs (1979, 1981), ultimately giving rise to the field of Experimental Pragmatics. As the theoretical pragmatic models continued to evolve, with a particular emphasis on psychological plausibility, this shift became necessary to empirically test both these models (Noveck and Sperber, 2004) and the growing array of pragmatic phenomena, meaning the events that elicit distinct pragmatic inferences according to their nature and the specific cognitive processes required for the inference (e.g., processing of irony, metaphors, speech acts, among others). Thus, the goal of experimental pragmatics is to determine, through complex experimental paradigms and neuropsychological tests, which cognitive functions are involved in pragmatic competence. In verbal communication, this competence corresponds to the ability to attribute mental states to a given speaker (Noveck and Sperber, 2004). This capacity is closely linked to Theory of Mind (ToM), also called a “mind reading” or “mentalizing” capacity, which allows us to infer other’s thoughts, beliefs and emotions (e.g., Canal et al., 2022; Frith and Frith, 2004; Muscatell et al., 2012). Nonetheless, even if ToM supports pragmatic competence, it cannot be completely reduced to it, since other cognitive functions play a role as well (Hagoort and Levinson, 2014). More specifically, the experimental approach of pragmatics has largely focused on the role of executive functions (including flexibility, working memory, inhibition, among others) on the processing of pragmatic information (Domaneschi and Bambini, 2020).

Although the field of clinical linguistics emerged in the 1800’s, clinical studies of pragmatic deficiencies were only first reported in the late 1960’s. Before that, pragmatic deficits were often overlooked, they lacked precise classification and were instead vaguely described as “high-level” linguistic processing impairments. The concept of pragmatic deficit was more formally structured in the late 1970’s and 1980’s, particularly through its association with right hemisphere brain lesions (Bambini and Bara, 2012). Accordingly, the “right hemisphere hypothesis” proposed a functional division of the brain. While the left side supported structural linguistic skills (e.g., phonology, syntax, semantics, etc.), the right one enabled creative and interpretative abilities. Given the lack of brain imaging methods at the time, clinical pragmatics primarily focused on stroke patients who, after lesioning the right side of their brains, exhibited deficient communicative competence despite their other linguistic abilities remaining intact. Rather than presenting typical aphasic symptoms, they had hindered “superordinate” language skills, such as the comprehension of abstract formulations (Bischetti et al., 2024). The main impacted pragmatic processes included discourse processing, interpretation of non-literal language, in which the unsaid must be interpreted, and mentalizing (Stemmer, 2008). In discourse processing, common symptoms of right-hemisphere damage (RHD), for example, difficulty maintaining a topic, identifying and extracting relevant information, interpreting emotional content from prosody, and ensuring discourse cohesion. These difficulties were associated with an inability to integrate information and draw inferences appropriately. Regarding non-literal language patients with RHD were able to produce and understand indirect speech requests, which require the listener to infer what is being asked, whereas other clinical groups, such as patients with traumatic brain injury (TBI) or schizophrenia, exhibited impairments in this ability. Some deficits in non-literal language processing in patients with RHD included difficulties with metaphor (also observed in patients with schizophrenia), irony (also present in patients with TBI), and humor interpretation (Stemmer, 2008). Concerning mentalizing deficiencies, while ToM skills were shown to be impaired in patients with frontotemporal dementia, Alzheimer disease and especially schizophrenia; RHD patients exhibited considerable heterogeneity. All this variability in pragmatic deficits concerns exclusively acquired deficits. However, similar mentalizing deficits are also observed in neurodevelopmental disorders, such as autism spectrum disorder (ASD). Individuals with ASD struggle with turn-taking, adapting their utterances to the interlocutor’s needs, and, most notably, inferring meaning from figurative language (Bischetti et al., 2024; Stemmer, 2008). Moreover, pragmatic deficiencies following traumatic brain injury have been shown to correlate with impairments in domain-general cognitive mechanisms, including working memory, attention, executive functions and social cognition (Rowley et al., 2017).

After more than 50 years of research in clinical pragmatics, there is a significant number of clinical populations that are impacted by pragmatic deficiencies. The prevalence varies from 40% to 80% across diverse neurodevelopmental, neurodegenerative, and even genetic disorders, from dyslexia, to multiple sclerosis, and Williams syndrome (see Bischetti et al., 2024 for a review). Accordingly, clinical pragmatics and the hypothesis of the role of the right hemisphere for pragmatic competence laid the groundwork for the emergence in the 2000’s of neuropragmatics (Bambini and Bara, 2012). Neuropragmatics can be defined as an interdisciplinary field, intersecting theoretical and experimental pragmatics, as well as neurolinguistics and neuropsychology. Although neuropragmatics draws concepts and methods from these adjacent fields, it cannot be fully encompassed within any of them. Unlike neurolinguistics, which focuses on the neural basis of language processing, or neuropsychology, which more broadly examines -through lesion studies- the brain processes that guide the human mind and behavior, neuropragmatics specifically focuses on understanding the neural mechanisms that enable the integration of linguistic material within a context (Bambini and Bara, 2012; Bischetti et al., 2024). It studies how the brain supports the comprehension and production of pragmatic information through the use of neuroimaging paradigms. In other words, it examines the neural circuitry and dynamics of pragmatic processing, namely inferring the meaning intended by a speaker and constructing utterances that are context-appropriate (Bambini, 2010; Hagoort and Levinson, 2014; Stemmer, 2008). In the following section, we will present and compare three theoretical models which can serve as potential neurocognitive models of pragmatic processing.

## 3 Models in neuropragmatics

Given the lack of a comprehensive neurocognitive model of pragmatic ability, this section aims to assemble the most relevant theoretical proposals that may serve as potential neurocognitive models of pragmatic processing, in order to better understand the neural mechanisms that support pragmatic inferences in naturalistic communicative situations. Some of them include a description of pragmatic processing both for comprehension and production in a dialogue.

### 3.1 A neurocognitive model of language integrating pragmatic processing

The first model we review does not exclusively focus on neuropragmatics; instead, it presents a broader neurocognitive framework of language processing, in which pragmatic processing is incorporated as a critical linguistic component. Through the years, the language neuroscientist Hagoort (2005, 2013, 2016, 2019) has extensively critiqued the classical Wernicke-Lichteheim-Geschwind (WLG) model of language processing (Geschwind, 1965; Lichteim, 1885; Wernicke and Eggert, 1874). According to the WLG model, Broca’s area (located in the left inferior frontal cortex) would be linked to language production, while Wernicke’s area (found in the left temporal cortex) would support speech comprehension. Hagoort argues that this model, which was primarily developed from studies of single-word processing, presents an oversimplified view of language processing. He points out that the functions attributed to Broca’s and Wernicke’s areas are not mutually exclusive: lesions in Broca’s area can also impair comprehension, and damage to Wernicke’s area can affect production. Consequently, Hagoort states that the WLG model is insufficient to account for the full complexity of language processing. Instead, he proposes that language comprehension and production rely on shared, distributed neural circuits (Hagoort, 2013, 2016) and develops the Memory-Unification-Control (MUC) model, which consists of three core cognitive mechanisms (Hagoort, 2005). The first component, Memory, refers to the linguistic knowledge acquired and consolidated through language learning. This knowledge is stored in neocortical memory regions and includes the fundamental building blocks of language: lexical items possessing distinct syntactic, phonological, and morphological properties. This is the only language-specific component of the model; the other two (Unification and Control) are considered domain-general cognitive systems (Hagoort, 2016). The Memory system alone is not sufficient, as linguistic competence extends beyond the mere retrieval and sequencing of lexical elements. This is where the Unification system comes into play: it enables higher level (sentence-level and beyond) meaning to be derived, incrementally. This mechanism is based on the Chomskian principle of generativity (Lyons and Chomsky, 1966), which states that humans possess the capacity to generate an infinite number of novel linguistic structures by combining stored lexical items. This combinatory capacity is represented in the Unification system, where retrieved lexical information is assembled into bigger structures, guided by the context (Hagoort, 2013). The Control system links language perception and production systems during social interactions, enabling interlocutors to, for example, select a “contextually appropriate language” and thus meet the demands of the conversational exchange. It is within these two last systems that pragmatic competence is incorporated into the MUC model (Hagoort, 2016), with the Unification system being guided by the context and the Control system supporting the regulation of language use, according to the socio-communicative demands of the interaction, since pragmatic ability is not only a linguistic ability, but also a social ability.

**FIGURE 1:**
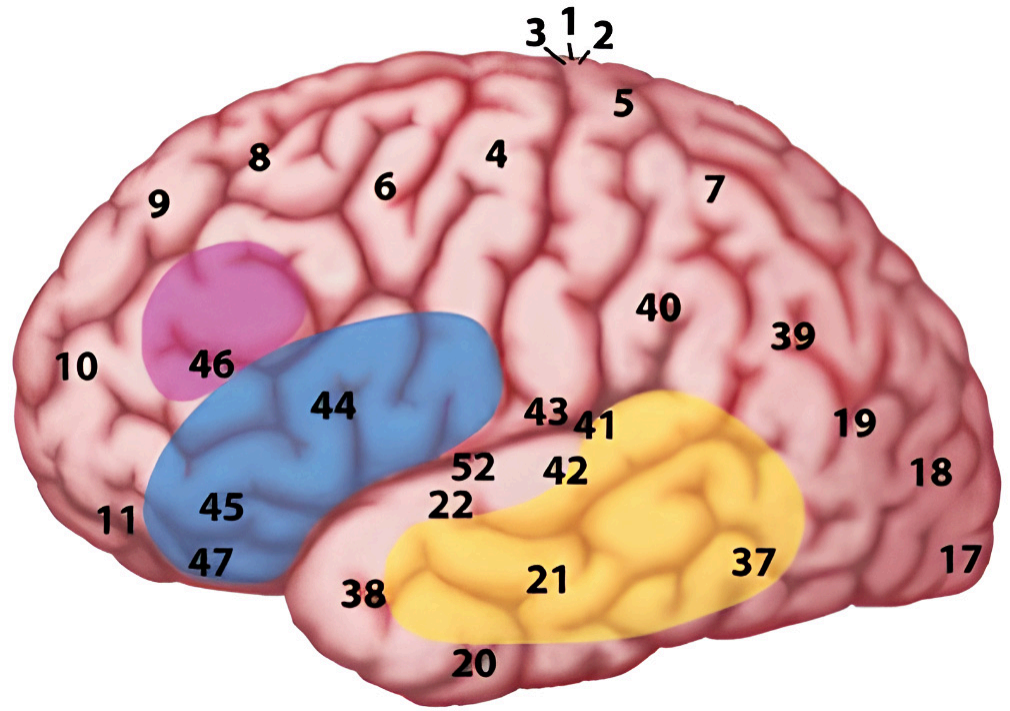
The MUC model of language Reproduced from Hagoort (2013). MUC (Memory, Unification, Control) and beyond. *Frontiers in Psychology*, *4*. https://doi.org/10.3389/fpsyg.2013.00416. Under the Creative Commons Attribution License (CC BY 4.0).

Each of the components of the MUC model has been associated with specific brain regions. Advances in neuroimaging, particularly fMRI, have significantly improved our understanding of where processes occur in the brain, providing millimetric precision in localizing neural hemodynamic activity (Price, 2012). Hagoort (2013) thus proposed a distributed network spanning across frontal and temporo-parietal regions, to serve as a neural substrate for his language model. Accordingly, the knowledge representations from the Memory component are supported by the temporal cortex and the angular gyrus in the parietal cortex; specific areas within these regions may be recruited depending on the type of knowledge employed. As for the Unification system, the retrieved memories are built into larger structures within frontal regions, particularly Broca’s region and adjacent areas (see Figure 1 for a schematic representation). The type of unified information determines the areas engaged: semantic, syntactic, and phonological processes primarily involve Broadman Areas (BA) 47 & BA 45, BA 45 & BA 44, and BA 44 & BA 6, respectively. Lastly, the Control regions (in charge of, for example, attention allocation and turn taking) are mainly sustained by the dorsolateral prefrontal cortex, as well as midline regions such as the anterior cingulate cortex and some parts from the parietal cortex (not included in Figure 1).

Hagoort’s model clearly steers away from the classical WLG model, to conceptualize language beyond single word processing. Although he does incorporate pragmatic elements into his framework, he does not provide detailed insights into how the pragmatic inferencing actually takes place. Hagoort (2019) did state, however, that part of the pragmatic processing relies on a dynamic interaction between temporo-parietal regions and the left inferior frontal cortex. This interaction enables the integration of knowledge (about the speaker, the context and the world) to determine the appropriate interpretation of an utterance (Hagoort, 2014; Van Berkum et al., 2008).

As early as 2013, Hagoort had pointed out the need for a speech acts theory to account for how speaker meaning is inferred. In order to bridge this gap, Bara and Tomasello’s models, examined in detail below, provide additional perspectives that yield a more comprehensive account of pragmatic processing in communicative situations.

### 3.2 Cognitive and neural models of pragmatic processing

**FIGURE 2:**
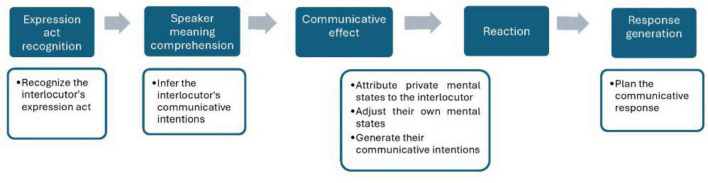
Steps to comprehend and generate a communicative act. Adapted from Bara (2010). *Cognitive Pragmatics: The Mental Processes of Communication*. MIT Press.

Bara’s Cognitive Pragmatics (2010) framework, which focuses on the interlocutors’ mental states, offers a useful protomodel with its “five stages of comprehension and generation of a communicative act.” Bara (2010) explains that although speaker meaning is constructed through processes that are parallel to each other, in order to analyze the structure of a conversation, it must be done by decomposing its basic components sequentially. Overall, the schema starts with a speaker generating an utterance, the listener in turn constructs a representation of their speaker meaning. As a result, the listener mental states linked to the conversational exchange might be modified, for example, if a belief expressed by the speaker is then integrated by the listener. Afterward, the listener must plan and generate their reply, based on their communicative intention. All of this occurs within what Bara (2010) calls the conversation game, which establishes a set of “metarules” that are specific to each exchange. These rules govern the structure of the conversation, guiding predictions and determining what is considered appropriate. From the main macrostructure, we can outline five steps that transpire within the listener’s mental states, see Figure 2 below for a schematic representation (Bara et al., 1993). In the first two steps of the model, we can clearly see the influence of Searle (1964, 1975); Grice (1968, 1975). The first step involves the listener perceiving the speaker’s communicative act as being purposefully directed toward them, with a clear intention from the speaker. This corresponds to the locutionary act, followed by the recognition of the illocutionary force, where the speech act is properly interpreted, which leads to the identification of the speaker meaning. This is achieved by attributing an intention to the speaker, guided by the conversation game and shared beliefs. In order to identify the communicative intent in the second step, the listener must make inferences that are “conversationally relevant”.

As shown in Figure 2, in the *expression act* (step 1), the listener constructs the speaker’s mental state from the locutionary act, followed by the *speaker meaning* (step 2), in which the listener identifies the intentions of the utterer (comprising indirect speech, if needed), next the *communicative effect* (step 3), which comprises the *attribution* (where the listener infers the speaker’s private mental states, including beliefs and intentions) and the *adjustment* (in which the listener’s mental states can be modified as a result), then the *reaction* (step 4), where the listener generates an intention to communicate and finally the *response* (step 5), in which the listener generates their own replying utterance, expressed through both linguistic and extralinguistic behaviors, which cannot be divided into smaller components, e.g., a smiling face (Bara, 2010).

Some of the limitations of Bara’s protomodel of pragmatics include the fact that it primarily focuses on the listener’s perspective (their interpretation of the speaker’s message, as well as their actions and intentions). Although the listener then changes roles in a conversation (from step 3 to 4) to become the speaker, the model fails to describe the neural processes underlying the pragmatic processing in a dialogue when two interlocutors take turns listening and speaking. Nonetheless, Bara also proposed a neurophysiological network to account for pragmatic processing (Walter et al., 2004). In 2004, Bara’s team collected an initial set of fMRI experiments which aimed to distinguish between individual and social intentions. Then, parallel to his theoretical description of how communication acts are produced and understood, by attributing an intention to the speaker, in (2010) Bara proposed the “Intentionality Network,” a dynamic model comprising four regions: the precuneus, medial prefrontal cortex (mPFC) and the left and right temporo-parietal junctions (TPJ). The purpose of this network is to explain the neural basis of various types of intentions, categorized according to their modality (individual vs. social) and their temporal dimension: present (communicative intention) or future (prospective social intention). According to the model, both the precuneus and right TPJ are needed to process any kind of intention. In contrast, the anterior paracingulate cortex and the left TPJ are exclusively recruited when processing social intentions. Moreover, the left TPJ is activated specifically for present social intentions, but not for prospective ones. Unfortunately, Bara did not establish clear anatomo-functional correspondence between the Intentionality Network and the other steps he outlined in a communication act, beyond the second step, where the speaker’s intention is identified.

### 3.3 Neuropragmatic model of speech acts

A decade after Bara’s proposal, Tomasello (2023) developed the first neuromechanistic model of communicative functions, based on Austin’s (1962), Searle’s (1975) speech act theory and Pulvermüller et al.’s (2014) Action Prediction Theory of Communicative Functions. The Action Prediction theory is a framework of cognitive processing which proposes that the knowledge of actions and perceptions is integrated through cell assemblies (action-perception circuits, APCs) that are connected through sensorimotor knowledge of actions. These serve as brain mechanisms to support various cognitive functions, including socio-communicative interactions. Pulvermüller et al. (2014) argued that action–perception links exist not only for hand and mouth movements with their visuo-acoustic perceptions, but also within human communication through “linguistic-articulatory action schemas.” Moreover, they proposed to expand the Action Perception theory to comprise linguistic-pragmatic mechanisms, considering that an utterance links to a different APC depending on the socio-interactive context in which it occurs, thus allowing the distinction between two speech acts. Specifically, Pulvermüller (2018) posited that through language learning we form action-perception circuits that are distributed across the cortex, which support cognitive-linguistic processes such as speech production and perception, as well as verbal working memory and even prediction. He explained that interpretating speech acts involves a complex interaction between sensori-motor, linguistic, and ToM brain activity. Consequently, he pointed out the need to carry out experimental research and develop neural models of language that account for these socio-communicative processes. Five years later, Tomasello (2023) did exactly this; after revising the neural research of speech act processing, he proposed a model, which we name the “Neuropragmatics of speech act model” after the title of the article in which it is described.

Tomasello’s (2023) model outlines how listeners access “speaker meaning” and generate corresponding action sequences, within a given speech act (e.g., a request followed by an acceptance or rejection). This structure closely resembles Bara’s overall stages of a communication act. Tomasello identified four fundamental traits of the pragmatic roles of speech acts: the *propositional content*, which corresponds to the utterance itself, the linguistic structure (locutionary act), the *communicative setting*, referring to the non-linguistic and physical context in which the utterance occurs, the *action sequence structure*, meaning the actions paired in response to a specific speech act, and lastly the *intentions and assumptions*, which include the beliefs shared by the interlocutors, the common ground, and other ToM aspects. The last two features determine what varies at the pragmatic level between different types of speech acts (e.g., distinguishing between a naming and a request speech act). Accordingly, the main premise of his model is that, neurally speaking, speech acts are supported by circuits that enable the processing of a speaker’s assumptions and intentions, as well as the typical action sequence that follows. As a result, pragmatic features that distinguish speech acts are processed differently in the brain: speech acts linked to actions activate the motor cortex, while other more socially complex, in turn, activate the ToM network. Furthermore, the predictions of the actions that the listener will take, following the speech act, are also neurally represented through more precise motor cortex activity.

Based on the evidence of verbal and gestural processing during the interpretation of communicative actions, Tomasello et al. (2019) identified the need to revise serial view of linguistic processing which conceptualize pragmatic processing as the final stage of the language processing cascade. Instead, he proposed that the socio-interactive information conveyed in communicative acts is processed instantly. He later confirmed this in his (2023) model, in which neuropragmatic processing occurs in parallel to other linguistic processes, namely pragmatic inferences take place simultaneously to the processing of semantic information. Accordingly, he presented evidence from several neuropragmatics studies showing early pragmatic processing of speech acts, with neural responses emerging as early as 150 ms after stimulus presentation (Tomasello, 2023). This efficient handling of linguistic actions is key for a proper turn-taking between interlocutors, and therefore, for effective socio-communicative interaction (Levinson, 2016).

**FIGURE 3:**
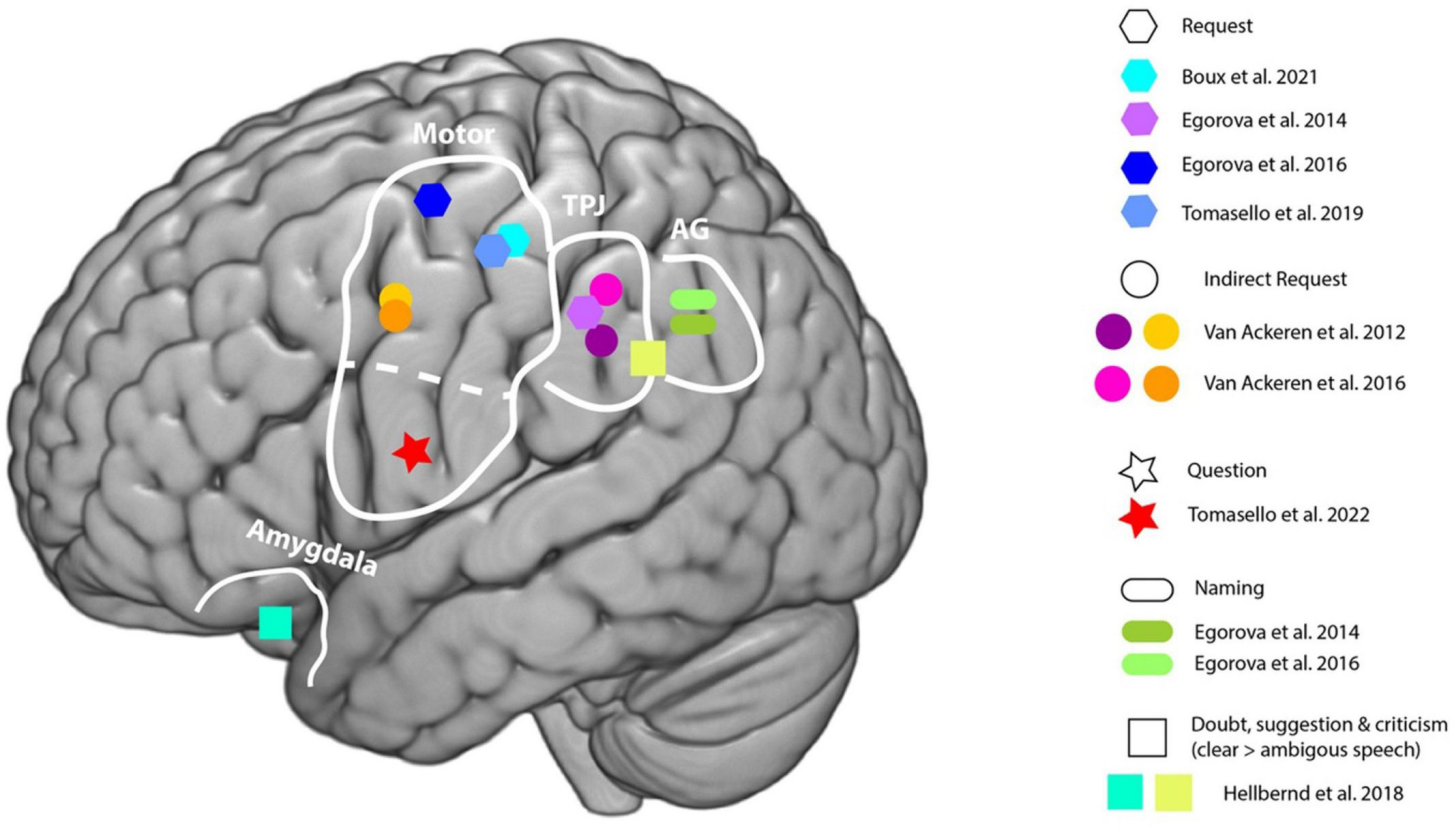
Brain regions in studies investigating different speech act types. Reproduced from Tomasello (2023). Linguistic signs in action: the neuropragmatics of speech acts. In *Brain and Language* (Vol. 236). https://doi.org/10.1016/j.bandl.2022.105203. Under the Creative Commons Attribution License (CC BY 4.0). Amygdala; Motor regions; TPJ, temporoparietal junction; AG, angular gyrus.

Currently, Tomasello’s (2023) proposal is the most up to date with the neuropragmatic research of speech acts. Nonetheless, the model presents an important gap: it cannot adequately account for other pragmatic phenomena beyond speech act processing, since it does not provide a functional distinction among the various types of pragmatic phenomena. Such distinction would be necessary to capture the potentially distinct neurocognitive processes involved, according to the nature of the pragmatic information being processed. To complement this theoretical proposal, Tomasello (2023) identified the brain regions associated with speech act identification and processing. Confirming predictions from the APC model, the comprehension of request speech acts activates the motor cortex (Egorova et al., 2016), given the following typical action sequence in response. This is also true for question speech acts, which also prompt an action sequence, namely the articulatory motor activity to reply to the inquiry. However, this activation pattern is not observed for naming speech acts, seen as there is not a logical action sequence. Instead, they place the focus on referential semantic information, which in turn activates the left angular gyrus. As communicative situations become more socially complex (i.e., greater shared intentions and obligations linked to specific speech acts, for example in a request compared to a statement) additional brain areas are recruited to process others’ mental states and beliefs. Once intentions and associated action sequences have been processed, the core ToM network (bilateral temporal junction) is then activated (Tomasello, 2023). This is the case for the comprehension of indirect speech acts (Bašnáková et al., 2014, 2015; van Ackeren et al., 2012, 2016), given that mentalizing is required to infer pragmatic meaning. Furthermore, beyond the ToM network, affective regions may also be recruited when processing communicative intentions involving criticism, suggestions, or doubt. See Figure 3 for a graphic representation of the brain regions activated during the processing of different speech act types.

Through the years, ToM has been associated both theoretically and neurophysiologically with pragmatic processing (e.g., Enrici et al., 2019; Sperber and Wilson, 2002). Concerning pragmatic inferences, which rely on assumptions about the interlocutors’ intentions, Hagoort (2019) argues that they depend on contributions from the ToM network. Moreover, he states that recognizing a speech act in accordance with the utterance context also engages mentalizing processes supported by this network. This perspective aligns with Bara’s neurospatial proposal and partially with Tomasello’s, as both emphasize the role of the right TPJ and the mPFC in inferring others’ beliefs and intentions. (2019). For a review on ToM and the brain substrates of direct and indirect speech acts comprehension, see Tomasello et al. (2025).

Even though the ToM network is indisputably involved in pragmatic processing (Bambini and Lecce, 2025; Cummings, 2015), as explained earlier, it cannot be completely reduced to this function, even when accompanied by core language processes (Bosco et al., 2018; Paunov et al., 2019). This conclusion is supported by Bendtz et al. (2022), who, through behavioral and neuroimaging assessments of linguistic, ToM, and pragmatic abilities, demonstrated that pragmatic processing, although partially overlapping with language and ToM networks, is neurocognitively distinct from both systems, in line with previous findings (Bosco et al., 2018; Paunov et al., 2019).

### 3.4 Comparison of current models in neuropragmatics

After presenting the existing theoretical proposals that serve as neurocognitive models of pragmatic processing, this section aims to identify their key insights and the gaps that remain to be addressed in order to guide the future development of Neuropragmatic models. Table 1 provides a summary of the main ideas presented in each model, as well as their respective contributions and limitations.

**TABLE 1:** Comparison of theoretical models of neuropragmatics.

References	Linguistic focus	Temporal dynamics	Cognitive components	Neural substrates	Contributions	Limitations
Hagoort (2013)	General language processing: sentence level	Immediacy principle: contextual information is used immediately	Memory: retrieval of linguistic information	Temporal cortex and angular gyrus	• Provides one-to-one cognitive-neural mechanism correspondences in language processing • Integrates pragmatic processing into neurocognitive model	• Does not explain in depth how pragmatic processing takes place • General view of pragmatics (variability of pragmatic phenomena not accounted for)
			Unification: combination of linguistic elements	Broca’s area and adjacent frontal cortex		
			Control: adaptation to context in social situations	Dorsolateral prefrontal cortex (dlPFC), anterior cingulate cortex, and parietal cortex		
Bara (2010)	Dialogue processing: beginning by listener’s role	Parallelism across linguistic processing stages	Expression act: speaker’s utterance	Intentionality network: • Temporo- parietal junction (TPJ) • Middle prefrontal cortex (mPFC) • Precuneus (PC)	• Developed detailed stages of how an expression act is understood and produced • Proposed neural substrates that support the inference of intention Approach to dialogue by taking into account changing of roles (listener becoming speaker)	• Missing neural processes for other aspects of pragmatics aside from intention, such as attribution and adjustment • Does not address variability of pragmatic phenomena Prioritizes the listener’s recognition of the speaker’s intention before acting
			Speaker meaning: listener identifies speaker’s intent			
			Communicative effect: attribution (listener infers speaker’s mental states) and adjustment (listener’s mental states are modified)			
			Reaction: listener’s intent			
			Response: listener’s utterance			
Tomasello (2023)	Dialogue processing: focused on speaker’s speech acts	Parallel phonological, lexical, syntactic, semantic and pragmatic processing (socio-interactive information is processed instantly)	Speech acts’ i. Propositional content: speaker’s utterance ii. Communicative setting: non-linguistic context iii. Action sequence structure: listener’s action response iv. Intentions and assumptions: shared knowledge between interlocutors	• ToM network: amygdala, TPJ and angular gyrus (to process intentions and assumptions) • Motor areas (for action sequence)	• First explicit model of neuropragmatics • Accounts for variability of speech acts (e.g., naming, request, question, etc.) • Approach to dialogue by taking into account changing of roles (listener is also a speaker)	• Does not account for other types of pragmatic phenomena • Prioritizes the speaker’s intentions and assumptions.

Although Hagoort’s (2013) MUC model presents clear cognitive-anatomical links and integrates pragmatics into general language processing, it does not provide a detailed account of how pragmatic functioning takes place. Bara’s (2010) model delves more in detail into pragmatic processing by specifying the steps that enable the production and comprehension of an expression act, incorporating a dialogue perspective and even proposing neural correlates required when inferring intention. Nonetheless, the proposed neural substrate is not described beyond intention processing, and it omits other pragmatic competences Bara details, such as adjustment or attribution. Moreover, Bara’s (2010) model primarily focuses on the listener’s perspective, starting with the listener’s recognition of the speaker’s intention from the utterance, before responding; in turn, Tomasello’s (2023) model emphasizes the speaker’s intentions and assumptions prior to intervening. Among the three models reviewed, Tomasello’s proposal is the only one explicitly framed as a neuropragmatic model. However, despite addressing a variety of speech acts, it cannot be adequately generalized to other pragmatic processes, since it does not functionally distinguish different pragmatic phenomena.

Furthermore, although, Bara (2010) discusses how metarules guide predictions within a conversation and Tomasello (2023) refers to predictions in terms of the expected actions a listener might take, none of the reviewed models explicitly integrate a conception of the brain as being proactive, meaning that it constantly generates expectations about incoming input and updates them by correcting prediction errors (Bar, 2009; Friston and Frith, 2015). This gap remains despite researchers advocating to incorporate the active inference perspective into neurolinguistic reasoning (Bouizegarene et al., 2024; Kuperberg and Jaeger, 2016). According to this view, higher level inferences, based on internal representations, are used to preactivate information on lower representational levels, and within this active generative framework, prediction is considered a main system guiding language comprehension.

In line with Hagoort’s (2019) “immediacy principle,” which proposes that contextual information is used without delay, as well as Tomasello’s (2023) account of early speech act integration, knowledge about the speaker and the context is thought to be used as soon as it becomes available. Similarly, Bara (2010) argues that the steps for intent construction occur in parallel rather than sequentially. These accounts all address aspects of the temporal processing of pragmatic information, particularly regarding its early and parallel nature. Nonetheless, they lack a comprehensive framework to explain the complete temporality of pragmatic processing in the brain, accounting for the different types of pragmatic phenomena and how they may impact the timing of the processing according to their complexity. Tomasello’s “Neuropragmatics of speech act model” is the most strongly supported by brain electrical research, allowing the author to conclude that speech acts are processed rapidly and in parallel to other linguistic information (e.g., semantic information; Egorova et al., 2013; Pulvermüller et al., 2014; Tomasello et al., 2019, 2022). Nonetheless, a deeper understanding of the temporal processing of pragmatic information is still necessary in order to determine how the variability of pragmatic phenomena affects its temporality. A non-exhaustive overview of the temporal dynamics of pragmatic inferencing is provided in Section 4.

### 3.5 New integrative framework of neuropragmatics

After comparing the different neuropragmatics model proposals, this section seeks to assemble the most important contributions of each model to incorporate into a future theoretical proposal of neuropragmatic functioning, considering their limitations and guided by the theoretical gaps that remain unaddressed. Despite Bara’s and Tomasello’s perspectives both adopting a dialogue perspective in their models, each one focuses primarily on one of the two interlocutors. Accordingly, an integration of both views for a future Neuropragmatic framework, could allow for a more comprehensive outlook of dialogue, taking into account the intentions, assumptions, and knowledge of both the speaker and the listener at the same time, both prior to and following the communication exchange. Likewise, the incorporation of Hagoort’s approach could provide the general language processing component that is inherently comprised in pragmatic processing.

As discussed in the previous section, a common limitation across the models reviewed is that they do not fully account for the variability across pragmatic phenomena. Specifically, they either adopt a general view of pragmatic processing [as in the proposals by Hagoort (2019), Bara (2010)] or focus on a specific type of pragmatic phenomena, such as speech acts [as in Tomasello et al.’s (2019)] proposal, despite integrating multiple speech act types). An alternative approach would be to understand pragmatic processing as being hierarchical, with functional distinctions among mechanisms according to the complexity of the phenomenon or even the skill of the interpreter. This approach is supported by the empirical findings of Bendtz et al. (2022), which show that the brain areas activated when processing indirect speech acts, particularly indirect replies, are modulated by individual differences in pragmatic competence. Using behavioral measures of both pragmatic comprehension and production (particularly, prosodic comprehension of requests in a communicative situation, and advanced audience design task requiring participants to adapt messages to different listeners), the researchers contrasted individuals with high versus low pragmatic skill in an fMRI study. Their results provide insight into the functional organization of neural mechanisms underlying pragmatic processing.

In Bendtz et al. (2022), the indirect speech act comprehension task activated common brain areas (including the bilateral inferior frontal gyrus, TPJ, anterior temporal lobe, medial superior frontal gyrus, dorsomedial prefrontal cortex - dmPFC-, and the mid and posterior middle temporal gyrus/superior temporal sulcus) regardless of the pragmatic skill level of the participant. These areas are typically associated with either language comprehension or ToM (Saxe and Wexler, 2005). Accordingly, Bendtz et al. (2022) proposed that the frontotemporal (language-related) regions support a top-down pragmatic processing, whereas the frontoparietal regions (linked to ToM) reflect perspective-taking and intention attribution. These activations likely correspond to a low-level (more general and basic) pragmatic processing, rather than a high level (more socially complex and cognitively demanding) inferencing. In contrast, participants with high pragmatic skills showed significantly greater activation in the dorsal precuneus and parietal cortex, which are not considered part of the core language or ToM regions (Schurz et al., 2014). Importantly, this activation did not correlate with behavioral measures of language processing (evaluated through vocabulary and lexical decision tasks) or ToM (assessed with a non-verbal mentalizing ability test), indicating the activation was specifically related to communicative inferences. Thus, Bendtz and colleagues suggest these two regions constitute a network for complex intention processing, specific to communicative settings. Additionally, these areas are thought to be linked with cognitive control, particularly higher-order pragmatic processes that support complex intention inferencing and attention attribution. Accordingly, Bendtz et al. (2022) put forward a *neurocognitive segregation* between domain-specific communicative inferences and domain-general ToM mechanisms. Moreover, they propose that the parietal cortex and the precuneus could both serve as biomarkers of pragmatic competence, consistent with previous empirical studies (Licea-Haquet et al., 2021; Powell et al., 2019).

Further neuroimaging studies are needed to better delineate the neuropragmatic network, particularly research addressing pragmatic phenomena beyond indirect speech. In this regard, the regions identified by Bendtz et al. (2022) are supported by previous fMRI reviews, comprising other types of pragmatic phenomena. Namely, Ferstl et al. (2008) delineate an “Extended Language Network” in which they highlight the role of the anterior temporal lobes and frontomedial cortex for language processing in context. Moreover, after examining different pragmatic phenomena including metaphors, irony, idioms, humor and sarcasm, Ferstl (2010) emphasizes the role of the dmPFC for non-automatic inferences, and the medial parietal cortex to represent discourse and update the situation model, defined as a representation of the listener’s or reader’s background knowledge that enables the integration of the message content (Van Dijk and Kintsch, 1983). Likewise, in their meta-analysis on the activation of brain areas associated with speech acts, metaphors, idioms, and irony, Reyes-Aguilar et al. (2018) conclude that pragmatic comprehension is enabled by a combination of classical language areas, notably bilateral perisylvian regions, as well as brain regions associated with social cognition, such as the medial prefrontal cortex. However, beyond the overall convergence of brain areas associated with pragmatic processing, an open question for a future integrative neuropragmatics framework is whether Bendtz et al. (2022) neurocognitive segregation proposal can adequately account for the findings of these reviews, and how. In other words, could the identified neuropragmatic network be organized hierarchically? For now, the boundaries of the neuropragmatic network remain broad, with pragmatic processing functioning as a bridge between brain areas implicated in socio-emotional, cognitive control, and linguistic processes. Furthermore, the temporal boundaries across pragmatic phenomena also remain unclear, as none of the models provide a theoretical account of the cognitive organization underlying the complex temporal dynamics of pragmatic processing. Accordingly, the next section aims to provide further detail on the diversity of these dynamics.

## 4 Temporal dynamics of pragmatic processing

During communication, pragmatic inferences are typically completed within milliseconds, in order for the turn taking to flow effectively. While behavioral measures can trace the outcome of this process, brain imaging techniques offer a significant advantage: they allow researchers to track cognitive mechanisms online, as they occur in real time (Van Berkum, 2009). While fMRI is highly informative in terms of spatial localization, its temporal limitations can be addressed with the use of electroencephalography (EEG), whose excellent temporal resolution can capture brain activity with high temporal precision. Accordingly, this technique makes it possible to test hypotheses on the timing of cognitive processes, that could not be addressed otherwise. Although not exhaustively, this next section synthesizes research using EEG to provide insights into the temporal dynamics of pragmatic inferencing. For a more extensive review and analysis of EEG research on pragmatic processing and its associated ERP components and oscillatory activity, see Canal and Bambini (2023).

### 4.1 Event-related potential studies in neuropragmatics

Event-related potentials (ERPs), that is, brain responses time-locked to a stimulus, are particularly useful within language studies, seen as they enable investigation of online neural processing. ERPs are waveforms with negative and positive voltage deflections that reflect how information flows through the brain, with amplitude reflecting neural activity at specific timepoints. These waveforms provide insight into the underlying internal cognitive mechanisms and their informativeness comes from how they differ across conditions (Luck, 2014). The precise temporal changes in neural processing that can be studied with ERPs can aid examine how context influences language comprehension in real time.

Over the past thirty years, ERP studies in neuropragmatics have helped characterize the main brain components associated with pragmatic processing, which can be broadly categorized into negative components and early and late positive components. To better illustrate them, selected examples for each ERP component are outlined below. It is important to note that the observed effects may vary across studies, not only as a function of the pragmatic phenomenon of interest, but also dependent on the evaluation task (e.g., passive perception, categorization, agreement rating, etc.). Moreover, the precise timing and scalp topography of the ERP components can also differ across studies, as a result of methodological differences. A summary of the reviewed studies is shown in Table 2, which provides additional details regarding the effects observed, the materials used, and the nature of the task (passive or active).

**TABLE 2:** Summary of event-related brain potentials (ERP) components associated with pragmatic processing.

ERP	Pragmatic phenomena	Significant differences between conditions	References	Material	Task	Interpretations
P100	Speech acts	Request > naming	Egorova et al., 2013	Audiovisual dialogues	Passive	Early, almost immediate, speech act detection Initial pragmatic processing Early LPC onset
			Tomasello et al., 2019	Visual: images and sentences	Active	
P200	Moral values	Value inconsistent > consistent	Van Berkum et al., 2009	Written sentences	Active	
	Irony	Irony > literal	Regel et al., 2011	Auditory discourse	Passive	
	Speech acts	Significant differences between speech acts	Gisladottir et al., 2015	Auditory dialogues	Active	
	Indirect replies	Indirect > direct replies	Zhang X. et al., 2021	Written dialogues	Passive	
P300	Idioms	Figurative > literal	Vespignani et al., 2010	Written sentences	Passive	Categorical (all or none, rather than probabilistic) predictions Speaker meaning updating
	Indirect replies	Indirect > direct replies	Zhang X. et al., 2021	Written dialogues	Passive	
N400	Indirect replies	Indirect > direct replies	Guo et al., 2023	Written dialogues	Passive	Parallel semantic and pragmatic processing Effort of non-literal meaning retrieval, Adapting meaning to context Difficulty linking meaning of incoming words in relation to the expectations set by previous context Incremental sensitivity to pragmatic information
			Zhang et al., 2023			
			Zhang X. et al., 2025			
	Metaphors	Metaphor > literal	De Grauwe et al., 2010	Written sentences	Active	
			Bambini et al., 2019			
			Forgács, 2020			
	Idioms	Literal > figurative	Vespignani et al., 2010	Written sentences	Passive	
			Canal et al., 2017			
	Scalar implicatures	Informative > underinformative	Nieuwland et al., 2010	Witten sentences	Passive	
		True > underinformative > false	Hunt et al., 2013	Visual: images and sentences	Active	
		Some > all	Spychalska et al., 2016			
	World knowledge	Violation > true	Hagoort et al., 2004	Witten sentences	Passive	
			Martin et al., 2015			
	Irony	Irony (positive) > literal	Foucart and Hartsuiker, 2021	Auditory discourse	Active	
			Baptista et al., 2018	Visual: images and sentences	Active	
			Caillies et al., 2019	Auditory discourse		
		Irony > literal	Caffarra et al., 2019		Passive	
	Humor	Humorous > Neutral	Coulson and Kutas, 2001	Written sentences	Passive	
			Feng et al., 2014		Active	
			Mayerhofer and Schacht, 2015		Passive	
	Speaker identity	Incongruent > congruent	Van Berkum et al., 2008	Auditory discourse	Passive Active	
			White et al., 2009	Written sentences		
	Moral values	Value inconsistent > consistent	Foucart et al. (2015b)	Written sentences	Active	
			Van Berkum et al., 2009			
Nref	Reference resolution	Ambiguous > clear referent	Van Berkum et al., 2003	Auditory discourse	Passive	Sensitive to referential accessibility Reflects referential ambiguity processing Extended N400 effort to derive meaning Anomaly detection (instead of N400)
			Nieuwland et al., 2007			
LAN	Humor	Mismatch > matching pronouns	Nieuwland, 2014	Written sentences	Active	
		Humorous > neutral	Coulson and Kutas, 2001	Written sentences	Passive	
			Canal et al., 2019			
	Metaphors	Metaphor > literal	Bambini et al., 2019	Written sentences	Active	
	Moral values	Value inconsistent > consistent	Kunkel et al., 2018	Written sentences	Active	
P600/ LPC	Irony	Irony > literal (right hemisphere)	Baptista et al., 2018	Visual: images and sentences	Active	Additional cognitive processing Supplementary linguistic processing, reverberation Late inferential processes Reanalysis and reinterpretation
		Irony (negative) > literal	Caillies et al., 2019	Auditory discourse		
		Irony > literal	Caffarra et al., 2019		Passive	
	Humor	Humorous > not humorous	Feng et al., 2014	Written sentences	Active	
			Marinkovic et al., 2011			
			Zheng and Wang, 2023			
	Idioms	Ambiguous idioms > literal	Canal et al., 2017	Written sentences	Passive	
	Metaphors	Metaphors > literal	Coulson and Van Petten, 2002	Written sentences	Active	
			De Grauwe et al., 2010			
			Bambini et al., 2016	Written sentences	Implicit	
			Weiland et al., 2014	Masked priming Auditory discourse		
	Indirect replies	Indirect > direct	Zhang X. et al., 2021	Written dialogues	Passive	
			Guo et al., 2023			
			Zhang X. et al., 2025			
	Speaker identity	Incongruent > congruent	Van Berkum et al., 2008	Auditory discourse	Passive Active	
			Foucart et al., 2015a			
	Moral values	Value inconsistent > consistent	Foucart et al., 2015b	Written sentences	Active Active	
			Van Berkum et al., 2009			
			Kunkel et al., 2018	Written sentences		

#### 4.1.1 Early ERP positivities

Several early positive ERP components have been linked to pragmatic processing, emerging within the first 300 ms following stimulus presentation. These include the P100, P200, and P300.

The P100, which peaks between 100 and 150 ms, has been shown to reflect early influences of pragmatic phenomena on sentence and utterance processing. For instance, Egorova et al. (2013) associated the P100 with a rapid speech act identification within a dialogue, in which the preceding context plays a crucial role by guiding this almost instantaneous processing. Similarly, Tomasello et al. (2019) found that brain responses to understanding different speech acts diverge as early as 130 ms. Changes in the predictability of the action following a speech act can modulate the timing of the ERP component, shifting its peak to around 200 ms when the subsequent action is unpredictable (Gisladottir et al., 2015). Although the P200 was originally linked to an initial semantic processing (Regel and Gunter, 2017), further studies have shown that it can also reflect the automatic allocation of resources for an initial pragmatic analysis, when interpreting indirect speech acts, namely indirect replies (Zhang X. et al., 2021). Moreover, this component has been observed in response to other types of pragmatic phenomena, including irony (Regel et al., 2011) and moral value violations (Van Berkum et al., 2009), also interpreted as an early onset to a later positive component. Lastly, the P300 component is thought to reflect information updating in working memory (Van Duynslaeger et al., 2007). Further neuropragmatics studies have associated it with pragmatic predictions when processing idioms, which are word strings with a figurative conventional interpretation (Vespignani et al., 2010), as well as with the integration of new pragmatic information into the previous mental model of the dialogue, namely when processing indirect replies (Zhang X. et al., 2021).

#### 4.1.2 Mid-to-late ERP negativities

Three main negative components have been shown to emerge when processing pragmatic information: the N400, the referential negativity (Nref), and the left anterior negativity (LAN).

The N400 is one of the most widely studied ERP components within language research. It is a negative deflecting potential that typically emerges around 300 ms and peaks at 400 ms after stimulus onset. Originally characterized in Kutas and Hillyard (1980), it was thought to be a marker of meaning processing. Further research demonstrated the amplitude of the N400 response is modulated by the degree of expectancy and the ease with which a word can be integrated into the preceding context (Kutas and Hillyard, 1984). Today, it is well-known that the N400 amplitude reflects the effort required to retrieve meaning from long term memory and adjust it to the available context (DeLong and Kutas, 2020). This interpretation is compatible with Hagoort’s MUC framework, which states -as explained in see section “3.1 A neurocognitive model of language integrating pragmatic processing” - that meaning is constructed through the retrieval of lexical knowledge from language-specific storage (Memory), followed by the combination of the retrieved elements in order to derive high-level meaning (Unification), and its subsequent adaptation (Control) to the socio-linguistic context (Hagoort, 2016). Accordingly, a given context can preactivate properties of an upcoming word, whether its nature is linguistic -sentential or discourse level-, non-linguistic, or even social -world knowledge and shared beliefs- (Kutas and Federmeier, 2011). Given that pragmatics is the study of language in context, we would then expect pragmatic processing to modulate the N400. Indeed, a growing body of research has shown that the resources required to draw pragmatic inferences influence the amplitude of the N400. This effect has been observed across a wide range of pragmatic phenomena, including indirect replies (speech acts in which the intended meaning is expressed implicitly); (Guo et al., 2023; Zhang et al., 2023, Zhang X. et al., 2025), metaphors (Bambini et al., 2019; De Grauwe et al., 2010; Forgács, 2020), irony (Baptista et al., 2018; Caffarra et al., 2019; Caillies et al., 2019), scalar implicatures (conversational implicature where using a scale term, e.g., “some,” implies stronger alternatives, e.g., “all,” are not true; (Hunt et al., 2013; Nieuwland et al., 2010; Spychalska et al., 2016), and humor (Coulson and Kutas, 2001; Feng et al., 2014; Mayerhofer and Schacht, 2015). Furthermore, N400 modulations have also been reported when processing world knowledge (factual information about the world; Foucart and Hartsuiker, 2021; Hagoort et al., 2004; Martin et al., 2014), speaker identity (Van Berkum et al., 2008; White et al., 2009) and moral values (principles about what is right and wrong); (Foucart et al., 2015b; Van Berkum et al., 2009) violations. In most of these cases, pragmatic information elicited larger N400 amplitudes, reflecting the increased cognitive effort required to retrieve and integrate contextually appropriate meaning. However, this pattern does not extend to idioms (Canal et al., 2017; Vespignani et al., 2010), whose meanings are culturally shared and easily recognized. Since idioms do not require a compositional interpretation (word for word), they tend to elicit smaller N400 amplitudes.

The Nref, or referential negativity, is another negative component associated with pragmatic processing, particularly linked to the resolution of referential expressions, that is, words or phrases (like names, pronouns, or noun phrases) that refer to specific entities. This negative-deflecting wave typically arises when listeners or readers must determine the most suitable referent in a context where the situation model is ambiguous. For example, the statement “Diana does not like Sophia because she is very resentful” is referentially ambiguous, given that the context does not clarify which character the pronoun “she” refers to. Accordingly, the Nref directly reflects a context-dependent pragmatic inference (Nieuwland, 2014; Nieuwland et al., 2007; Van Berkum et al., 2003, 2007). The Nref effect usually emerges in frontal regions, between 300 and 400 ms after stimulus presentation and can last up to 1,200 ms (Van Berkum, 2009).

Lastly, the LAN is a negative-going long-lasting component with a characteristic anterior scalp distribution. It is most commonly associated with syntactic incongruities (Molinaro et al., 2011). Nonetheless, growing evidence suggests the LAN can also be modulated by pragmatic processing. Specifically, it has been observed when interpretating literary metaphors (Bambini et al., 2019), humor (Canal et al., 2019; Coulson and Kutas, 2001) and moral transgressions (Kunkel et al., 2018), emphasizing its role to detect socio-pragmatic incongruities.

#### 4.1.3 Late ERP positivities

Two late positivities are commonly observed during pragmatic inference: the P600, which typically peaks around 600 ms after stimulus onset, and the Late Positive Component (LPC) -also called the Late Positive Potential (LPP)-, which reaches its maximum amplitude later on (e.g., 800–900 ms after stimulus presentation). However, due to their temporal overlap and similar topographical distribution (Canal and Bambini, 2023), these components are rarely analyzed separately in pragmatic contexts. Therefore, they will be discussed here as a single late positivity. As was the case for other components (e.g., LAN), the P600 was initially thought to reflect syntactic processing (Kaan et al., 2000; Osterhout and Holcomb, 1992); distinguishing it from the semantic anomalies originally associated with the N400 (Kutas and Hillyard, 1984). Years later, Coulson and Van Petten (2002) reported a late positivity associated with metaphor comprehension, which they interpreted as additional recovery and integration of semantic information. Since then, numerous studies have identified late positivities linked with metaphor interpretation (Arzouan et al., 2007; Bambini et al., 2016; De Grauwe et al., 2010; Weiland et al., 2014). Following these studies, current interpretations of the P600 and LPC extend beyond syntax, suggesting that these components reflect a continued reanalysis of non-literal meaning, serving as markers for inferential processing, and indicating the additional cognitive effort required to resolve pragmatic ambiguities. Accordingly, these late positive components have been reported across an extensive range of pragmatic phenomena. Irony processing, for instance, has largely been associated with the P600 component (Baptista et al., 2018; Caffarra et al., 2019; Caillies et al., 2019). Moreover, both the LPC and P600 have been linked to the interpretation of indirect speech acts, more specifically indirect replies (Guo et al., 2023; Zhang X. et al., 2021, 2025), in addition to the processing of idioms (Canal et al., 2017), moral values (Foucart et al., 2015b; Kunkel et al., 2018; Van Berkum et al., 2009) and speaker identity (Foucart et al., 2015a; Van Berkum et al., 2008) violations. Lastly, these late positivities have been associated with both the resolution of incongruity (P600) and the subsequent appreciation or elaboration phase (LPC) when interpreting humor (Feng et al., 2014; Marinkovic et al., 2011; Zheng and Wang, 2023).

### 4.2 ERP findings in the context of current models of neuropragmatics

As evidenced in Table 2, there is substantial variability in the temporal dynamics of pragmatic processing. Across the diverse pragmatic phenomena presented, processing effort and time demands seem to vary according to the nature and associated complexity of the pragmatic phenomena at hand. This variability likely relates to the cognitive processes required to integrate the specific pragmatic phenomenon, as well as to the brain areas supporting these processes (e.g., TPJ, mPFC, precuneus, among others; see section “3.5 New integrative framework of neuropragmatics” for more detail). While previous neuroimaging studies have identified key regions implicated in pragmatic processing and have outlined the overall temporal signatures, no current neuropragmatic model provides a neurocognitive framework capable of fully accounting for this observed temporo-spatial variability.

As discussed previously, Tomasello’s proposal has the most ERP evidence among the reviewed models. Nonetheless, the integration of the different theoretical frameworks could lead to a more comprehensive account of pragmatic processing: incorporating basic language processing principles from Hagoort’s proposal, as well as the perspectives of both interlocutors in a dialogue, each one emphasized in Bara and Tomasello’s models. Furthermore, the neurocognitive segregation proposal from Bendtz et al. (2022) provides insights to conceive a comprehensive functional organization of pragmatic phenomena in the brain; in which domain-specific resources would support more effortful and slower pragmatic processing, engaging higher-level brain areas, while domain-general resources would account for faster, less effortful, and lower-level pragmatic processing. Although this distinction may appear reductionist at first, the assignment of pragmatic phenomena to either type of resource is in fact highly nuanced and likely depends on multiple cues, including the available contextual information, interindividual differences in pragmatic and executive abilities, and speaker identity.

Moreover, research has shown that inferences about a speaker’s intentions and other pragmatic cues can influence predictions during language processing (Lowder and Ferreira, 2019; Ryskin and Nieuwland, 2023). In line with this, the early ERP components reviewed in this section (Egorova et al., 2013; Tomasello et al., 2019; Vespignani et al., 2010), which current theoretical accounts interpret as reflecting an “ultra-rapid” processing of pragmatic information, suggest that predictions in conversation could allow interlocutors to preactivate linguistic information, thus facilitating pragmatic processing. Likewise, previous research shows that probabilistic predictions can temporally modulate pragmatic processing, as reflected by ERP components such as the N400 and the P600 (Kuperberg, 2013, 2016). Nevertheless, current neuropragmatic proposals have yet to effectively incorporate an active inference perspective (Bouizegarene et al., 2024; Friston and Frith, 2015), which could significantly contribute to the development of an integrative neuropragmatic framework. The next section further details the different cues that influence pragmatic processing and introduces emerging techniques and methodologies that are likely to advance both empirical findings and the accompanying theoretical frameworks in neuropragmatics.

## 5 Current and future directions in neuropragmatic research

After detailing what is currently known about neuropragmatics, from the origins of the field to the available theoretical models as well as current findings on temporal dynamics of pragmatic processing, this final section discusses what remains unknown. Particularly, what is missing from the available models, followed by the gaps in the field and the future methodological approaches that could help resolve pending questions.

Beyond clinical studies (reviewed in see section “2.3 Bridging experimental and clinical methods: the birth of neuropragmatics”), individual differences also play a crucial role in shaping pragmatic skills. Previous research in behavioral pragmatics has demonstrated that individual differences modulate pragmatic performance (Ryzhova et al., 2023; Schuster et al., 2023). Particularly, social skills (as measured by the Autism-Spectrum Quotient) and cognitive abilities such as inhibition, abstract reasoning, and working memory can all influence pragmatic performance in various pragmatic tasks. These include perspective taking (Loy and Demberg, 2022), solving implicatures in reference games (Mayn and Demberg, 2022), interpreting redundant utterances (Ryzhova et al., 2023), adapting to speakers’ uncertainty expressions (Schuster et al., 2023), and humor processing (Loy and Demberg, 2024). The integration of individual factors in neuropragmatics studies is quite rare, although some evidence shows that variables such as working memory (Canal et al., 2019; Zhang X. et al., 2021), socio-pragmatic skills (Bendtz et al., 2022; Nieuwland et al., 2010), and even IQ (Kazmerski et al., 2003) can modulate neural responses during pragmatic inferencing and inform on how pragmatic processing is organized in the brain (Bendtz et al., 2022). This has been observed in tasks involving humor processing, metaphor comprehension, and indirect speech interpretation. In the future, neuropragmatic research should account for inter-participant variability, seen as these individual factors are embedded in the broader context, significantly impact pragmatic inferences, and can help us better understand the mechanisms underlying pragmatic processing.

Another factor that greatly influences pragmatic performance is the multilingual context in which the inference takes place. Few studies have focused on how pragmatic information is processed in the brain, when comprehending a second language (L2) or a foreign accent. Research on L2 neuropragmatics has investigated the integration of pragmatic phenomena such as world knowledge (Romero-Rivas et al., 2017), speaker identity (Foucart et al., 2015a), metaphors (Miller et al., 2025), or moral values and emotions (e.g., Foucart et al., 2015b). Altogether, these studies suggest that L2 speakers can efficiently use the available context given their heightened sensitivity to pragmatic information, as demonstrated by an earlier positive brain response (Foucart et al., 2015a).This is in line with the Shallow Structure Hypothesis (SSH), developed by Clahsen and Felser (2018), according to which L2 speakers are mainly guided by pragmatic and surface-level information, while having a reduced sensitivity to morpho-syntactic information, compared to L1 comprehension. Other studies suggest that integrating pragmatic information in an L2 requires additional cognitive effort and this may vary depending on the type of pragmatic phenomenon involved. For example, Zhang X. et al. (2025) recently published an EEG study on indirect speech act processing with an L2 reading task. They found that non-native participants show a delayed processing of indirect replies, which they interpreted as a cognitive resource limitation. Despite these advances, much remains to be understood about L2 and even more about L3 neuropragmatics. Accordingly, multilingual neuropragmatics could become a prominent field for future research. For a thorough review of the current state of L2 neuropragmatics, see Citron (2023). Meanwhile, research on foreign-accented neuropragmatics is even more rare than in L2 contexts. Some authors have explored the impact of foreign-accented speech on the processing of world knowledge violations (Foucart and Hartsuiker, 2021) and irony (Caffarra et al., 2018, 2019). Overall, these studies have identified a hindered pragmatic inferencing capacity, associated with shallower pragmatic processing and disrupted anticipatory mechanisms, likely resulting from the disfluencies of the foreign-accented speech. Nonetheless, our knowledge of how different accents impact neuropragmatics processing is still limited; from the variety of pragmatic phenomena that remain unstudied, to the ways in which accent variability may modulate this impact according to factors such as familiarity, associated preconceptions and social stereotypes, or proximity to the native accent.

Even though within language processing, there is an increasing interest in analyzing oscillatory brain dynamics (for reviews see Meyer, 2018; Prystauka and Lewis, 2019), that is, how the brain waves synchronize across time and space, there is currently no comprehensive framework linking oscillatory dynamics to the cognitive mechanisms that enable pragmatic processing. Brain oscillations arise from rhythmic fluctuations in neural activity, typically characterized in frequency bands: delta (1–2 Hz), theta (4–7 Hz), alpha (8–12 Hz), beta (15–30 Hz), and gamma (30–200 Hz). Oscillatory changes across these bands are associated with specific cognitive computations that support language processing (Prystauka and Lewis, 2019). A key advantage of oscillatory analysis compared to ERPs is that oscillations provide a continuous signal comprising both phase-locked (to the stimulus) and non–phase-locked activity. Hence, it can provide a broader view of the neural underpinnings of pragmatic processing, to complement ERP analysis (Gisladottir et al., 2018). Existing evidence shows that processing pragmatic phenomena such as world knowledge violations (Hagoort et al., 2004), irony (Akimoto et al., 2017; Regel et al., 2014), idioms (Canal et al., 2017; Rommers et al., 2013), humor (Canal et al., 2019), and speech act identification (Gisladottir et al., 2018) can be associated with decreases in either alpha or beta bands, or with increases in either gamma or theta bands. Given the scarcity of time–frequency studies on pragmatic processing and the richness of oscillatory analysis, this line of work could develop into a particularly fruitful research direction.

Another prominent field for future research can come from more realistic paradigms of everyday life language use. Traditional neurocognitive paradigms in language research rely on controlled experiments designed to isolate a specific stimulus or task in order to determine their impact on brain activity. Unfortunately, these paradigms often largely differ from everyday language use, thus limiting their generalizability to real-life contexts. Consequently, a current challenge in language research is the development of more naturalistic paradigms, which would broaden the range of linguistic phenomena to analyze and enhance the ecological validity of the results (Li et al., 2024; Zhang Y. et al., 2021). Following the strive for naturalistic experimental designs, interactive paradigms provide the opportunity to steer away from assessing single participants in isolation and instead study the natural dynamics of real dialogue (Zada et al., 2022). Hyperscanning has emerged as a technique to study participants’ brain activity as they engage in real-time conversations. It allows simultaneous measurement of neural processes using, for example, two EEG systems (Bridwell et al., 2018; Kerr et al., 2024) or two fMRI scanners (Speer et al., 2024; Zada et al., 2025). Hyperscanning, as a technique, is still in its infancy; as a result, many methodological and theoretical issues remain unsolved [for reviews, see (Babiloni and Astolfi, 2014; Nam et al., 2020)]. It is highly probable that this and other interactive techniques (such as brain-to-brain coupling) will continue to evolve, particularly with the rise of large language models (Zada et al., 2022). These methodologies could thus become quite promising within neuropragmatics, given the insight they provide into the highly context-dependent nature of interactive communication.

Moreover, current neuroimaging studies have mainly focused on correlational approaches, which cannot inform on the functional contributions of a given brain region. To move past purely correlational studies toward causal links between brain anatomy and pragmatic function, brain lesion studies -once crucial for the emergence of both neurolinguistics and neuropragmatics- are becoming increasingly useful again. Parallelly, brain stimulation studies that simulate virtual lesions, including non-invasive techniques (e.g., transcranial magnetic stimulation and transcranial direct current stimulation) and invasive approaches such as intracranial electrical stimulation combined with intracranial EEG, provide additional causal evidence (Ntemou et al., 2023; Siddiqi et al., 2022). In recent years, there has been a growing interest in brain stimulation techniques to study pragmatic processing (e.g., Boux and Pulvermüller, 2023; Martín-Luengo et al., 2023). For a comprehensive review of the use of neurostimulation techniques in pragmatic language research, see Kurada (2025), which highlights the role of specific brain regions in distinct pragmatic phenomena. Additionally, in order to incorporate the variability of pragmatic phenomena and move beyond the exclusive questions of *where* or *when* pragmatic processing takes place to address *what* kind of pragmatic information is represented, neuroimaging and stimulation studies could benefit from the integration of multivariate approaches. These include representational similarity analyses, which measure the (dis)similarity of neural response patterns, and classification approaches including linear discriminant (Ufer and Blank, 2024). Importantly, these approaches require an accompanying cognitive rationale to help interpret *how* said pragmatic information is represented.

Lastly, amid the growing development of artificial intelligence (AI) tools, numerous opportunities arise to facilitate and enhance scientific research (Zhang Y. et al., 2025). Particularly neuropragmatic research can benefit from AI services (e.g., Microsoft Azur or Amazon Web Services), supporting various stages, from the generation of linguistic materials to the processing of neurolinguistic data through Natural Language Processing (NLP), for a review see Supriyono et al. (2024). Deep learning and artificial neural networks could also aid in the complex modeling of neuropragmatic processing (Goldstein et al., 2024). Furthermore, advances are being made to study and enhance the pragmatic performance of human-machine interactions, involving both conversational and embodied agents (see Chai, 2023; Dombi et al., 2022).

## 6 Conclusion

Throughout this review, a historical overview of neuropragmatics has been presented, following its origins from far-side pragmatics (with seminal proposals by Grice and Austin & Searl), through its evolution into cognitive and experimental pragmatics, which in turn led to the development of clinical pragmatics and ultimately, the birth of neuropragmatics. Moreover, this paper examined both theoretical and empirical findings that have shaped the understanding of pragmatic inferences within communication. Although no model yet accounts for both the neural and cognitive mechanisms underlying pragmatic processing, three model proposals were reviewed. Hagoort’s and Bara’s proposals provide approximations, respectively, through a general neurocognitive framework of language processing and a cognitive pragmatics schema, accompanied by a spatial conception of intentionality. Tomasello’s “Neuropragmatic model of speech acts” is, to date, the only model explicitly framed as a model of neuropragmatics. However, it focuses exclusively on speech act processing, leaving a broad range of pragmatic phenomena unaddressed. Furthermore, none of the available models of neuropragmatics effectively provide a theoretical proposition of how the cognitive processes underlying different pragmatic phenomena unfold over time and how their complexity modulates processing dynamics. Accordingly, we identified key gaps in current theoretical models and outlined possible directions towards a more integrative neuropragmatic framework.

Why would different pragmatic phenomena require different supporting neural mechanisms? Pragmatics processing is an umbrella capacity that encompasses diverse phenomena, ranging from humor and irony interpretation to the resolution of referential ambiguity and the integration of information related to moral values or world knowledge. Each of these phenomena involves distinct mental processes (including anomaly or incongruity detection, prediction, or updating), while also relying on shared abilities, such as attentional allocation and the use of contextual cues. Evidently, the nature of the contextual information itself can vary; it may be syntactic (as in referential resolution), semantic (as in metaphor comprehension), or socio-cultural (as in the interpretation of idioms). Given this variability across pragmatic phenomena, it is natural for the underlying neural substrate to differ not only in their spatial distribution, but also in their temporal dynamics, depending on the cognitive effort they require, which in turn varies with experience and the complexity of the specific phenomenon (Drijvers et al., 2025). This was reflected in the diversity of ERP components identified in this review, across the vast array of pragmatic phenomena. This variability illustrates why proposing a unified comprehensive model of neuropragmatic processing is quite challenging and would require great flexibility. Nonetheless, developing a separate neuropragmatic model for each phenomenon, as Tomasello did for speech acts, does not entirely resolve this issue. Although it provides a valuable contribution, this approach overlooks the flexibility inherent to pragmatic processing in the brain.

Future models in neuropragmatics should strive to encompass an integrative view of the field, incorporating domain-general and domain-specific spatio-temporal networks in the brain (Bendtz et al., 2022), as well as the corresponding theoretical proposal describing the cognitive organization underlying pragmatic processing. Likewise, this comprehensive view should also capture the variability of supporting mechanisms across the wide range of pragmatic phenomena. Furthermore, to develop a comprehensive framework of pragmatic processing, researchers must work collaboratively to overcome methodological heterogeneity arising from differences in task demands, sample characteristics, or linguistic materials. Interdisciplinarity is at the heart of neuropragmatics, placed at the crossroads between neurolinguistics, neuroscience, and experimental pragmatics (Bischetti et al., 2024). Accordingly, pragmatic processing itself is an aggregation of linguistic, cognitive, and socio-emotional mechanisms that enable the integration of speaker’s communicative intentions. The versatile nature of pragmatics is what makes it challenging, but it is also the source of its richness and promise for future research. With the rapid development of new technologies, paradigms, and methods in communicative and interactive contexts, neuropragmatics is set to remain a prolific research domain for many years to come.
